# Four weeks of exercise regimen for sedentary workers with rounded shoulder posture: a randomized controlled study

**DOI:** 10.1590/1516-3180.2022.0257.R1.06072022

**Published:** 2022-09-12

**Authors:** Ozge Ece Gunaydin, Ersen Ertekin, Gurkan Gunaydin

**Affiliations:** I PhD, PT. Assistant Professor, Department of Physical Therapy and Rehabilitation, Faculty of Health Sciences, Aydın Adnan Menderes University, Aydın, Turkey.; II MD. Associate Professor, Department of Radiology, Faculty of Medicine, Aydın Adnan Menderes University, Aydın, Turkey.; III PhD, PT. Assistant Professor, Department of Physical Therapy and Rehabilitation, Faculty of Health Sciences, Aydın Adnan Menderes University, Aydın, Turkey.

**Keywords:** Posture, Shoulder, Exercise, Photogrammetry, Taping, Scapular correction, Stiffness, Elastography

## Abstract

**BACKGROUND:**

Rounded shoulder (RS) posture causes neck and shoulder pathologies. Mechanical correction taping (MCT) is often incorporated into postural corrective therapies; however, its effects on muscle stiffness are unclear.

**OBJECTIVE:**

We investigated the effect of MCT with different tape fabrics, along with exercise, on upper trapezius and pectoralis minor muscle stiffness and the posture of sedentary workers.

**DESIGN AND SETTING:**

A randomized controlled study was performed at Aydın Adnan Menderes University, Aydın, Turkey.

**METHODS:**

The study included 39 workers with RS posture. Two intervention groups (performance tape: PT and classic tape: CT) were taped twice a week and administered a home exercise program for 4 weeks. The control (C) group performed only home exercises. RS was measured using an acromion-testing table (AT), stiffness using shear wave elastography ultrasound, and shoulder angle (SA) using a smartphone application at baseline and 4 weeks. Time and group interactions were determined using 3 × 2 mixed analysis of variance.

**RESULTS:**

Intragroup analyses revealed a significant main effect of time on AT distance (η^2^ = 0.445) and SA (η^2^ = 0.325) in the PT and C groups (P < 0.05) and left upper trapezius stiffness (η^2^ = 0.287) in the CT and C groups (P < 0.05). In the post hoc analyses, no difference was noted between the groups from baseline to 4 weeks (P > 0.05).

**CONCLUSION:**

Scapular MCT added to postural exercises did not show any difference between the intervention groups and controls in terms of muscle stiffness and posture in sedentary workers.

## INTRODUCTION

Posture is defined as the sequence of body parts required to maintain musculoskeletal balance.^1-3^ Rounded shoulder (RS) posture is a postural disorder in which the line of gravity shifts anteriorly. This shift causes the head and shoulder positions to be inconsistent with the vertical line of the body, leading to “poor” posture.^1,2,4^ Poor posture of the head and shoulders is associated with the risk of increased muscle load, degenerative disc disease, back pain, and chronic shoulder pathologies.^3,5^ RS posture also develops with alignment impairments in the scapular position. A protracted and anteriorly tilted scapula creates excessive stress and increases muscle tone.^6,7^ According to the literature, the muscles with the highest increase in tone are the upper trapezius and pectoralis minor.^2,3^

Prolonged sitting in a static position increases poor posture. Therefore, the possibility of developing poor posture is especially concerning in desk workers. Working in a static environment exacerbates the RS posture over time, causing^8,9^ loss of function^10^ and work efficiency in workers.^11^

Several different applications used for posture correction have been described in the literature.^12,13^ Among these, scapular mechanical corrective taping with elastic tape is a popular technique.^14-16^ In a study using corrective taping, it was shown that this application significantly improved shoulder posture.^9^ In a systematic review, scapular corrective taping was suggested to improve scapular posture in both healthy individuals and patients with shoulder problems.^17^

Muscle stiffness is considered valuable information in the diagnosis of neck and shoulder problems.^18^ The shear wave elastography method has high reliability and provides objective data by converting the stiffness of the muscle in a localized area into numerical data.^19^ In the literature, there is no study explaining the effectiveness of scapular mechanical correction taping (MCT) on shoulder muscle stiffness using the shear wave elastography method. Therefore, it remains unclear whether corrective taping regulates muscle stiffness in the long term.

## OBJECTIVE

Our primary hypothesis was that MCT applied in addition to exercise would decrease the stiffness of the upper trapezius and pectoralis minor muscles and fix shoulder posture in workers with RS deformity. The secondary aim of this study was to determine whether different tape fabrics affected the application results.

## METHODS

### Participants

This study was conducted at a university hospital with 45 sedentary workers aged 18–34 years. Participants were included if they had RS posture with reference to a prior screening using lateral acromion-testing table distance (AT distance) measurement. Based on the results of this test, individuals with a result of 3 cm or more were considered to have RS posture, as shown in a previous study.^9^,^20^ The participants were excluded if they had a musculoskeletal system injury in the past 6 months or any neurologic or orthopedic disorder or cervical radiculopathy, received physical therapy in the last 6 months, participated in professional sports, or had an allergic skin reaction to the tape material. All procedures were explained to the participants, and written informed consent was obtained. Ethical approval for the study was obtained from the Ethics Committee of Aydın Adnan Menderes University, Faculty of Health Sciences Non-Interventional Clinical Investigations (No. 92340882-050.04.04; protocol: 2018/14), dated April 25, 2018.

### Study design

This study was a randomized controlled, single-blinded clinical study. The participants were randomized using a computer-assisted randomization method. For this process, the sequence generator available at “www.random.org” was used. The participants did not have any information about the groups formed or which group they belonged to. Therefore, a single-blinded study was performed. The study included two intervention groups, classic tape (CT, n = 15) and performance tape (PT, n = 15), and one control group (C, n = 15). Data were collected using a smartphone-based photographic analysis application, Dr. Goniometer (CDM S.r.L, Milano, Italy), for shoulder angle (SA) and using shear wave elastography for muscle stiffness. Elastographic evaluations of the participants were performed by a radiologist at the Radiology Department of the same university. Worker height (cm), weight (kg), and age (years) were recorded as demographic information.

### Sample size

Assuming that a strong degree of effect size (f = 0.5) was obtained for the difference between the three groups as a result of the power analysis conducted a priori in the direction of hypothetical expectations with reference to a similar study,^9^ at least 42 individuals (14 for each group) were required to obtain 80% power with 95% confidence.

### Intervention procedures

At the first visit, all the participants underwent baseline measurements. Immediately after the first visit, the tape was applied to the participants in the CT and PT groups. CT is a classical corrective tape made with a regular corrective fabric. PT is a new form of corrective tape developed by the same brand for the same purpose although with a different fabric. There is a difference in weaving between the different fabrics of the tape, allowing them to be thicker or thinner. The tapes of the participants in these groups were reapplied twice a week for a total of 4 weeks. The workers continued their home exercise program for 4 weeks. The workers in the control group performed only the home exercise program. Measurements were repeated in the same order at the end of 4 weeks. The tape was removed during the final measurement.

### MCT application

**Figure 1:**
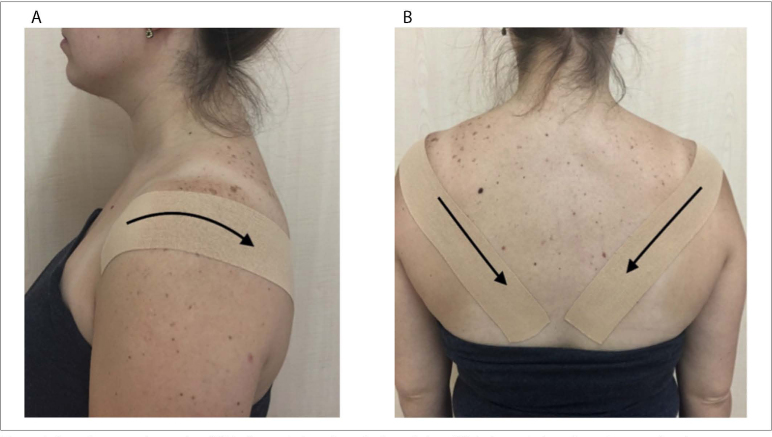
Scapular correction taping. (**A**) Taping technique from the lateral view. (**B**) Taping technique from the posterior view.

The tape application was performed by a physiotherapist with 12 years of experience, who was a certified corrective taping practitioner. First, an I-shaped tape was measured and cut in a personalized manner for each participant. The anchor of the tape was applied on the anterior aspect of the glenohumeral joint without any tension as the participant sat upright. Subsequently, the participant was asked to retract the scapula bilaterally. While the participant was maintaining this position, the tape was diagonally applied to the inferior border of the scapula with 50–75% tension and the last anchor was applied with no tension.^14,21^ The tape was applied to left and right shoulder girdle. The same taping technique was applied to the CT and PT groups using different tape fabrics (Figure 1).

### Exercise program

The home exercise program prescribed to all participants consisted of basic postural exercises. These exercises were performed to strengthen the scapular muscles and provide healthy posture. Participants followed the home exercises as indicated in Table 1.

**Table 1.** Exercise program

	Exercise	Explanation	Intensity
**‘W’ wall slides**	At the edge of the wall, when the back is in full contact with the wall, the arms are first opened to the side and bent by the elbows (making a W), dragging upward on the wall without interrupting the arm contact with the wall, and subsequently returning to the initial W position.	3 sets × 15 Twice a day 4 weeks
**Shoulder retraction**	Elbows are bent at 90° with arms adjacent to the body. In this position, the shoulder blades are squeezed for 5 s and held close to each other and then loosened. Concurrently, care should be taken not to pull up the shoulders. It is recommended to perform this exercise in front of the mirror if possible.	3 sets × 15 Twice a day 4 weeks
**Backward shoulder rolls**	While the arms are adjacent to the trunk, and the elbows are bent, the shoulders are rolled up first, then backward, and downward. The shoulder is required to make a full circle movement. This exercise is continued for 2 min.	3 sets × 2 min Twice a day 4 weeks

These exercises were provided to both the intervention groups and control group.

### Outcome measures

#### RS assessment (AT distance)

In the supine position, the acromion was palpated and marked, and the vertical distance between this point and the testing table was measured using a ruler. The measurements were recorded in centimeters. The reliability of this test was demonstrated in a previous study (intraclass correlation coefficient = 0.95).^22,23^ In this study, individuals with a measurement result of > 3 cm were considered to have RS.^9^

#### Postural angle assessment (Dr. Goniometer application)

**Figure 2:**
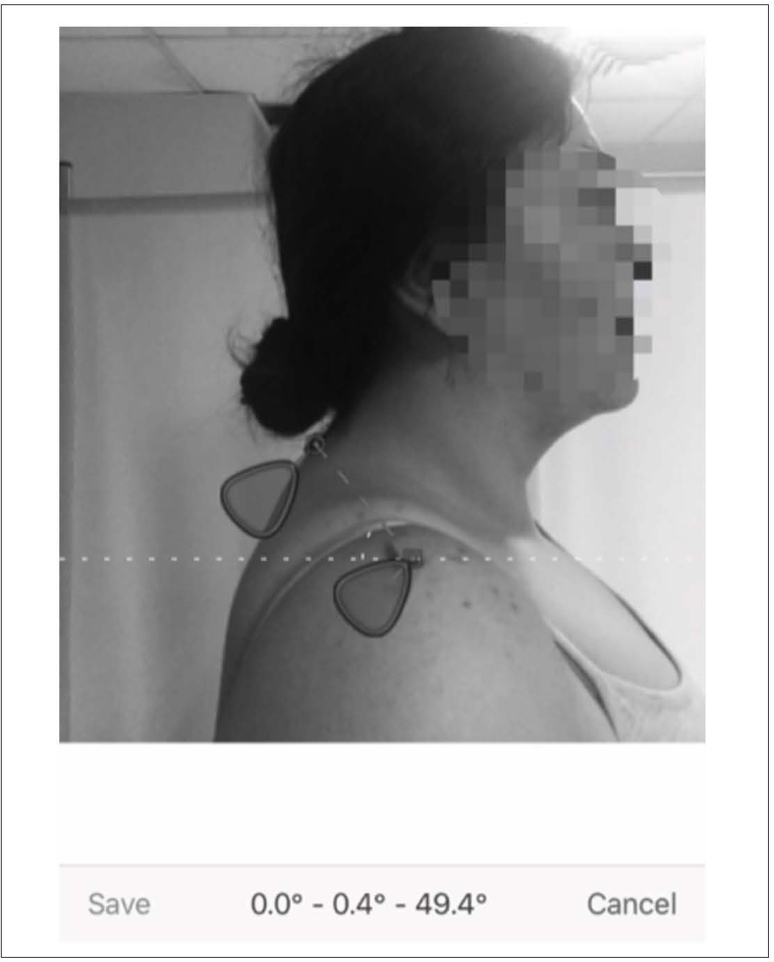
Shoulder angle assessment using Dr. Goniometer

Photogrammetry is the most commonly used noninvasive postural measurement because it eliminates possible exposure to harmful radiation during the radiographic method and does not require printing of photographs. Grading is performed by marking the reference bone points and measuring the distance or angle between the specified points.^1^ Before the measurement, the reference bone points (the acromion and seventh vertebra) were marked using a pen or a reflective marker to be clearly observed on the photograph. The camera was set up to take a photograph of the participant from the right lateral side. The participant was subsequently asked to lean forward and backward three times to relax and assume a comfortable standing position. While the participants breathed properly and stood still, a point was marked on the wall directly opposite the participant’s eye level to maintain posture. After taking the photo, the cursors on the application screen were adjusted, and the desired angle was recorded (Figure 2). Validity and reliability studies of this smartphone application were conducted, and the intraclass correlation coefficient value was found to be 0.92.^24^

#### SA

This angle occurs at the point where the line joining the seventh cervical spinous process with the acromion of the shoulder intersects the horizontal line. This angle decreases as the shoulder protraction increases.^1,2^

#### Shear wave elastography

Evaluations were performed using a Samsung RS80 Ultrasound Device (Gyeonggi-do, Republic of Korea), and the stiffness of the upper trapezius and pectoralis minor muscles was examined. A 4 cm transducer and a 9 MHz linear probe were used for imaging. The ‘musculoskeletal present’ setting was used. The probe was placed parallel to the muscle fibers. At least 10 consecutive measurements were performed for each muscle, and the median was obtained. For standardization, measurements with a quality factor of 0.4–1.0 reliability measurement index were used in each muscle. Measurements below the 0.4 reliability measurement index are omitted. The test for the upper trapezius muscle was performed while the participant was in a sitting position with the hands resting on the thigh. For the pectoralis minor test, the participant was in the supine position with the arms resting on both sides. The muscle shear modulus was recorded in kilopascals (kPas), considering that each assessment was taken at the same point, and the level of probe pressure was equal.^25^

### Data analysis

The Statistical Package for Social Sciences (SPSS) (version 22.0; SPSS Inc., Chicago, Illinois, Unites States) was used for data analysis. For the three groups (CT, PT, and C) at two different times of assessment (at baseline and 4 weeks), time and group interactions were determined using 3 × 2 mixed analysis of variance. For significant P values, Bonferroni corrections were used for post hoc analysis. Effect sizes were interpreted using partial eta squared (η^2^) and Cohen’s d. Partial eta squared (η^2^) values were accepted as follows: 0.01 = small, 0.06 = medium, and 0.14 = large.^26^ Cohen’s d values were accepted as follows: < 0.20 = small, 0.21–0.50 = small and medium, 0.50–0.80 = medium and large, and > 0.80 = large.^27^ Significance level was set at P < 0.05.

## RESULTS

**Figure 3:**
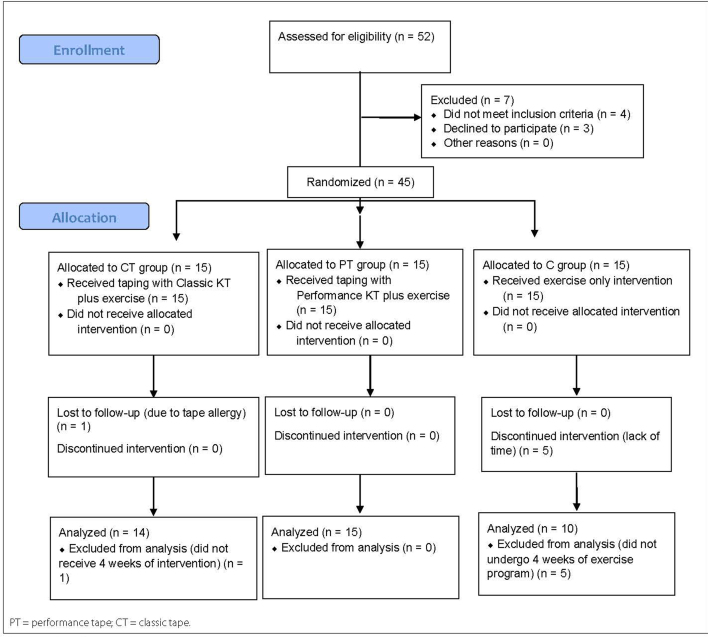
Flow diagram.

A total of 52 sedentary workers were assessed, of whom four did not meet the inclusion criteria and three declined to participate. Forty-five eligible individuals were randomized into three groups. Six participants dropped out for various reasons. Final analyses were conducted using data from 39 individuals (Figure 3).

The demographic characteristics of the 39 participants with RS posture (PT group, n = 15; CT group, n = 14; C group, n = 10) included in the study are shown in Table 2. The mean age of the participants was 23.3 ± 3.6, 22.5 ± 4.1, and 23.3 ± 5.4 years for the PT, CT, and C groups, respectively. The mean height was 164.06 ± 6.3, 164.7 ± 5.07, and 163.3 ± 4.96 cm for the PT, CT, and C groups, respectively. The mean weight was 63.3 ± 12.6, 63.2 ± 13.9, and 57.1 ± 6.3 kg for the PT, CT, and C groups, respectively (Table 2).

**Table 2.** Demographic characteristics of the groups

Groups	Variable	Min	Max	Mean ± SD
**PT** **(n = 15)**	Age (year)	19.00	32.00	23.3 ± 3.6
	Weight (kg)	48.00	95.00	63.3 ± 12.6
	Height (cm)	145.00	170.00	164.06 ± 6.3
**CT** **(n = 14)**	Age (year)	18.00	33.00	22.5 ± 4.1
	Weight (kg)	48.00	86.00	63.2 ± 13.9
	Height (cm)	158.00	176.00	164.7 ± 5.07
**C** **(n = 10)**	Age (year)	19.00	34.00	23.3 ± 5.4
	Weight (kg)	43.50	65.00	57.1 ± 6.3
	Height (cm)	155.00	170.00	163.3 ± 4.96

Min = minimum; Max = maximum; SD = standard deviation; PT = performance tape; CT = classic tape; C = control.

The effect sizes of the mean differences, 95% confidence intervals, P values of the SA, and stiffness values of the upper trapezius and pectoralis minor muscles at baseline and 4 weeks are shown in Table 3. In intragroup analyses, a significant main time effect was noted in AT distance (F [1, 36]: 28.860, P < 0.001, partial η^2^: 0.445) and SA degrees in the PT and C groups (F [1, 36]: 17.348, P < 0.001, partial η^2^: 0.325) and in left upper trapezius stiffness in the CT and C groups (F [1, 36]: 14.462, P = 0.001, partial η^2^: 0.287) (P < 0.05). However, in the post hoc analyses, none of the evaluated parameters showed differences among the groups from baseline to 4 weeks (P > 0.05) (Table 3).

**Table 3.** Baseline and fourth week results of the group

Parameter	Group	Baseline	At 4 weeks	Difference (95% CI)	P value	Cohen’s d
		Mean ± SD	Mean ± SD			
**SA** (degrees)	PT	61.25 ± 8.93	68.19 ± 9.10	6.94 (2.53–11.35)	**0.003**	0.76
	CT	63.84 ± 9.35	65.84 ± 5.21	1.99 (−2.57–6.56)	0.382	0.26
	C	57.91 ± 9.39	66.09 ± 5.40	8.18 (2.78–13.580)	**0.004**	1.06
**AT** **distance** (cm)	PT	6.10 ± 1.21	5.05 ± 0.80	1.05 (0.49–1.61)	**0.001**	1.02
	CT	6.40 ±1.28	5.55 ± 1.15	0.850 (0.27–1.43)	**0.005**	0.69
	C	6.48 ±1.40	5.58 ± 1.26	0.900 (0.21–1.59)	**0.012**	0.67
**Trapezius Stiffness Right** (kPa)	PT	43.38 ± 13.79	37.55 ± 12.72	5.83 (−0.78–12.43)	0.082	0.43
	CT	41.64 ± 14.44	38.94 ± 19.01	2.70 (−4.14 to 9.54)	0.429	0.15
	C	38.98 ± 9.73	34.71 ± 12.96	4.27 (−3.82–12.36)	0.292	0.37
**Trapezius Stiffness Left** (kPa)	PT	46.24 ± 17.26	42.41 ± 13.43	3.83 (−1.82–9.49)	0.177	0.24
	CT	47.94 ± 16.82	40.52 ± 16.08	7.42 (1.58–13.27)	**0.014**	0.45
	C	42.59 ± 17.33	33.83 ± 8.64	8.76 (1.84–15.68)	**0.015**	0.63
**PecMinor Stiffness Right** (kPa)	PT	18.83 ± 14.26	15.13 ± 10.28	3.69 (−5.23–12.62)	0.407	0.29
	CT	22.38 ± 11.65	21.33 ± 13.22	1.05 (−8.19–10.29)	0.819	0.08
	C	26.99 ± 31.13	17.12 ± 12.95	9.87 (−1.06–20.80)	0.075	0.41
**PecMinor Stiffness Left** (kPa)	PT	15.42 ± 8.39	13.51 ± 8.39	1.91 (−11.70–15.51)	0.778	0.22
	CT	20.63 ± 12.60	19.88 ± 12.38	0.75 (−13.33–14.83)	0.915	0.06
	C	34.40 ± 49.49	18.22 ± 14.81	16.18 (−0.48–32.84)	0.057	0.44

SD = standard deviation; SA = shoulder angle; PT = performance tape; CT = classic tape; C = control; kPa = kilo pascal; P < 0.05.

## DISCUSSION

To our knowledge, this is the first randomized controlled study to investigate the effects of MCT on muscle stiffness. After 4 weeks of mechanical scapular correction taping application, the results showed that the RS posture significantly decreased in all groups; however, the SA was corrected only in the PT and C groups. Improvement in muscle stiffness was only observed in the left upper trapezius in the CT and C groups. No superiority was observed among the intervention groups and controls in terms of the evaluations.

Deformities in soft tissue stiffness, muscle activity, and bone alignment may cause alterations in scapular and humeral movement. Consequently, several conditions, such as impingement, rotator cuff disease, joint instability, and capsulitis, may occur.^15,28^ To prevent these shoulder problems during repetitive overhead movements, a stable scapula and coordinated activity of the scapulohumeral muscles are required. Impairments in scapular movements during activities are associated with shoulder pain because they cause excessive stress and microtrauma to soft tissues.^29,30^ Scapular taping is one of the most useful methods for increasing joint stability by providing biomechanical realignment of the scapula and glenohumeral joint during several activities. With this technique, which is applied using an elastic therapeutic tape, both the normal healing of soft tissues and the stability of the joints are supported without restricting the range of motion.^14^ In a systematic review, it was suggested that scapular corrective taping could be used to improve scapular posture in both healthy individuals and patients with shoulder problems.^17^ A placebo-controlled study also reported a reduction in supine AT distance measurement for RS posture with mechanical scapular correction taping.^9^ On the contrary, in a study investigating the acute effect of bilateral scapular mechanical correction technique by using corrective and rigid tapes on posture in university students with significant shoulder protraction, no significant effect was reported.^31^ Similarly, Gulpinar et al. used the mechanical correction technique with both corrective and rigid tapes to determine the acute effect on RS posture.^32^ They positioned the glenohumeral joint in external rotation using the mechanical correction technique. They found an increase in the total range of motion of the shoulder in acute measurements; however, no change in posture was noted.^32^ The use of this technique is controversial in the literature. In these studies, posture was generally measured immediately after taping. In contrast, we evaluated the long-term effects in our study. The results were inconsistent with different fabricated tapes. The SA did not change in the CT group; however, it significantly increased in the PT group. Intergroup analyses also showed that no superiority of scapular MCT on the SA over the controls was noted. Since the numerical increase in the SA was mostly in the control group, we cannot conclude that the angular change was due to taping. We postulate that the exercise program had the necessary effect.

Overactivation and stiffness of the upper trapezius and pectoralis minor muscles associated with weakness of the lower trapezius and rhomboid muscles may cause a relatively protracted shoulder and disrupt normal posture.^33,34^ In desk workers, upper trapezius stiffness was shown to increase as the inclination angle of the head changes.^6,35,36^ Like the upper trapezius, proper tension–length relationship and pectoralis minor stiffness are also associated with optimal scapular posture.^9,37^ Average stiffness values of the upper trapezius were calculated as follows: 40–47 kPa in the sitting position and 0° neutral cervical position and 60–83 kPa in 50° cervical forward flexion in healthy participants using the shear wave elastography method.^38^ Average pectoralis minor stiffness was also measured as 12.7 ± 3.6 kPa in healthy individuals in another study.^39^ In our study, in the baseline measurements, upper trapezius stiffness measured in the neutral position was found to be between 40 and 47 (±9–17) kPa in all groups, whereas pectoralis minor stiffness ranged between 15 and 34 (±14) kPa, similar to the study by Zhang et al.^38^ In this situation, scapular taping may be a useful technique to reduce the symptoms. The tape can adjust the muscle activity through proprioceptive feedback.

It was reported that upper trapezius activity decreased and proprioception improved in individuals who underwent scapular corrective taping.^15^ In our study, instead of reducing the muscle stiffness using a direct application, we expected a decrease in stiffness that may occur secondary to posture correction with proprioceptive feedback. After 4 weeks of scapular MCT, a significant decrease in left upper trapezius muscle stiffness in the CT and C groups was noted; however, no intergroup superiority was observed. The pectoralis minor stiffness in the C group also numerically decreased; however, this was not statistically significant. The decrease in the stiffness level of the upper trapezius muscle in both the CT and C groups indicated that this decrease was not caused by MCT. Thus, we can argue that a 4-week home exercise program regulated muscle stiffness by improving shoulder posture. In addition, although the participants included in this study had an RS posture, the initial stiffness values were similar to those in studies on healthy individuals. This may be another reason why the decrease in stiffness levels was not significant. While there are studies showing the effectiveness of exercise on postural angles in the literature, none have evaluated muscular properties using shear wave ultrasonography. Kim et al. showed that McKenzie exercises were effective in increasing the craniovertebral angle and decreasing RS posture.^40^ Likewise, in another study, similar exercises were performed while MCT was added to the intervention group. In that study, it was shown that the greatest improvement in craniovertebral angle occurred in the exercise group, and a well-planned exercise program was concluded to help improve posture.^41^ In the present study, the exercise program prescribed to all participants included simple postural corrective exercises. The fact that no difference was noted between the groups showed that even a 4-week posture training with simple exercises could achieve the same effect.

Our study had some limitations. First, we performed pre-screening using supine AT distance measurement instead of angular measurement, while similar studies used angular measurements as the inclusion criteria of the participants. We recommend using postural angle data obtained by the photogrammetric method as an inclusion criterion in future studies. Second, a healthy group was not included in the study for comparison. Thus, in future studies, the baseline measurements can also be compared with those of healthy individuals.

## CONCLUSION

Scapular mechanical correction using corrective taping in addition to a postural exercise program was not found to be effective for muscle stiffness and posture in workers with RS posture. Different fabrics of tape materials did not result in significant changes. Therefore, prescribing corrective postural exercises will be more effective for the treatment of muscle stiffness that develops secondary to postural disorders. Based on our study results, we do not recommend the use of scapular MCT with the expectation of corrective and muscle stiffness regulatory effects in the long term.
